# Triplex DNA: A new platform for polymerase chain reaction – based biosensor

**DOI:** 10.1038/srep13010

**Published:** 2015-08-13

**Authors:** Yubin Li, Xiangmin Miao, Liansheng Ling

**Affiliations:** 1 School of Chemistry and Chemical Engineering, Sun Yat-Sen University, Guangzhou 510275, P. R. China; 2 School of Life Science, Jiangsu Normal University, Xuzhou 221116, PR China

## Abstract

Non - specific PCR amplification and DNA contamination usually accompany with PCR process, to overcome these problems, here we establish a sensor for thrombin by sequence - specific recognition of the PCR product with molecular beacon through triplex formation. Probe A and probe B were designed for the sensor, upon addition of thrombin, two probes hybridized to each other and the probe B was extended in the presence of Klenow Fragment polymerase and dNTPs. The PCR amplification occurred with further addition of Taq DNA Polymerase and two primers, the PCR product was recognized by molecular beacon through triplex formation. The fluorescence intensity increased with the logarithm of the concentration of thrombin over the range from 1.0 × 10^−12^ M to 1.0 × 10^−7^ M, with a detection limit of 261 fM. Moreover, the effect of DNA contamination and non - specific amplification could be ignored completely in the proposed strategy.

The polymerase chain reaction (PCR) technology is an essential tool for gene research, because the exponential amplification efficiency enables the detectionof small numbers of nucleic acid^1^. To extend the scope of PCR to high sensitive detection of protein, Sano *et al.* established the immuno - PCR (IPCR) method by combines antibody recognition and PCR amplification^2^,^3^,^4^,^5^,^6^, which could lead to about 100 - 10000 fold increase of sensitivity over the conventional ELISA for protein^7^,^8^,^9^. Owing to the exponential amplification and high sensitivity of PCR, it has been widely applied in detection of gene mutation^10^,^11^,^12^,^13^, pharmacogenetics^14^,^15^, gene expression analysis^16^,^17^,^18^, recognition of methylation specific loci^19^,^20^,^21^, protein^22^,^23^,^24^,^25^,^26^, detection of metal ion^27^ and constructing of DNA nano - structures^28^. However, non - specific amplification is inevitable during the process of PCR amplification. Therefore, the sensitivity and selectivity of PCR technique is controlled by detection method for PCR product. There are mainly five kinds of methods. Firstly, gel electrophoresis, it is generally cumbersome and time - consuming, which has been successfully used in the IPCR^29^,^30^,^31^. Secondly, Detection electrochemical signal of guanine nucleobase of PCR product after separation^32^. Thirdly, detection of PCR products with the TaqMan probes^33^. Fourthly, PNA or LNA modified molecular beacon through displacement hybridization^34^. Fifthly, intercalative molecules, such as Ethdium bromide and SYBR Green I etc., their fluorescence increase upon addition of double - stranded DNA, but they cannot distinguish PCRproduct from non - specific amplification product such as primer - dimers^35^. It is still a challenge to sequence - specifically recognize the duplex structure of PCR product.

Homopurine·Homopyrimidine duplex DNA can be sequence - specifically recognized by homopyrimidine Oligonucleotide (or homopurine Oligonuceotide) through triplex formation^36^,^37^. Study of triplex DNA mainly focus on following aspect: firstly, biological function of triplex DNA, such as their effects on the process of gene translation^38^, DNA transcription, replication and cleavage^39^,^40^; Secondly, effect of hole transport^41^,^42^,^43^,^44^; Thirdly, pH controlled switchable conformation change between duplex DNA and triplex DNA^45^,^46^,^47^,^48^; Fourthly, sequence - specific recognition of double - stranded DNA^49^,^50^,^51^,^52^,^53^,^54^; Fifthly, biosensing for other molecules^55^,^56^,^57^,^58^,^59^,^60^. However, there had little biosensor that based upon triplex formation and PCR amplification.

To overcome the drawback of PCR - based biosensor and explore new direction for the study of triplex DNA, here we developing a novel biosensor for protein by combining the amplification property of PCR and sequence - specific recognition ability of triplex formation for DNA duplex. As a proof of concept, here a sensitive fluorescent sensor for thrombin is being established with molecular beacon based upon triplex formation and PCR.

## Results and Discussion

**Figure 1:**
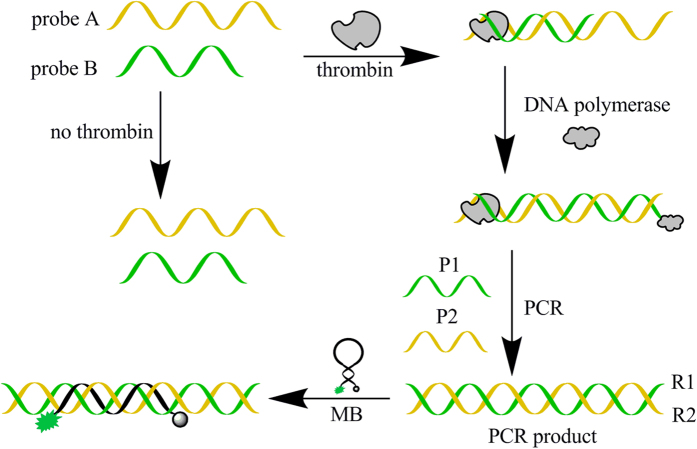
Scheme of the biosensor for thrombin.

**Figure 2:**
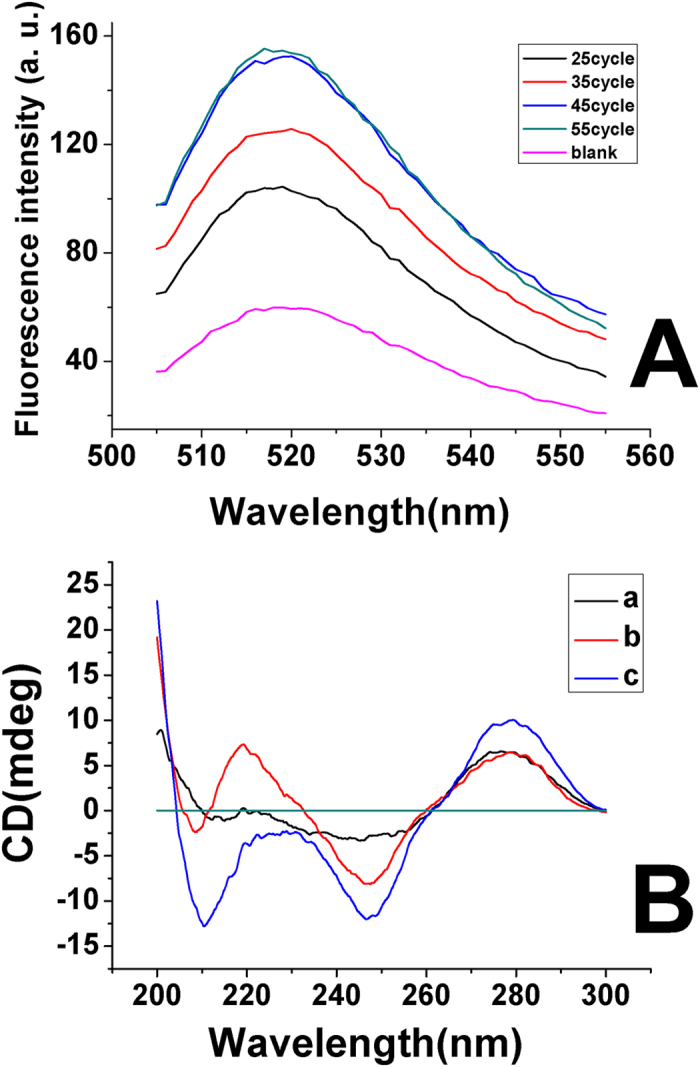
(**A**) Fluorescence spectroscopy of molecular beacon after hybridization with different cycle of PCR product. The PCR amplification was carried out under the condition of 30 s denaturation at 94 °C, 30 s of annealing temperature at 60 °C, and 10 s of extending temperature at 72 °C. MB: 200 nM, spermine: 0.4 mM, pH: 5.0. (**B**) Circular dichroism (CD) spectroscopy of Oligonucleotides under different conditions. a. MB: 1.0 × 10^−5^ M; b. R1·R2: 1.0 × 10^−5^ M; c. Mixture of 1.0 × 10^−5^ M MB and 1.0 × 10^−5^ M R1·R2. Spermine: 0.4 mM, pH: 5.0.

The scheme of the biosensor is shown in Fig. 1. Oligonucleotide 5′-AAT ACC CGA TTG CAG TAC GAC TCT C*CA CAA GCC* TTT TTT TTT TTT TTT TTT TTT TTT TTT TTT TT**G GTT GGT GTG GTT GG** -3′ (probe A) and Oligonucleotide5′-CG**A GTC CGT GGT AGG GCA GGT TGG GGT GAC T**AA AGA GAA GGA AGA GAA GAA GAA AGA AAA GAA AAA GTT TTT TT*G GCT TGT G*-3′ (probe B) are designed for the sensor, the underlined sequence are aptamers for thrombin, the T_32_ in probe A and T_7_ in probe B are designed as spacer. Oligonucleotide5′-AAT ACC CGA TTG CAG TAC GAC TC-3′ (P1) and 5′-GAG TCC GTG GTA GGG CAG GT-3′ (P2) are designed as primers. Upon addition of thrombin, the red and italic sequences of two probes hybridize to each other and the probe B is extended in the presence of Klenow Fragment polymerase and dNTPs. Then PCR amplification is carried out with further addition of dNTPs, Taq DNA Polymerase and two primers. Green sequence of Probe B is homopurine strand, it is designed to create homopurine•homopyrimidine double strand during the process of PCR amplification, which can be recognized by molecular beacon with the sequence of 5′-6-FAM-GTG GAG TTT CTC TTC CTT CTC TTC TTC TTT CTT TTC TTT TTC CTC CAC-BHQ-1-3′ (MB) through triplex formation^49^,^50^,^51^,^52^,^53^,^54^. As shown in Fig. 2A, the fluorescence intensity of PCR product and molecular beacon was only about 60 in the absence of thrombin, it increased to about 155 upon addition of 1.0 × 10^−7^ M thrombin. These results confirmed the probability of the proposed strategy.

The numbers of PCR cycle affect the fluorescence intensity of sensor. As shown in Fig. 2A, the fluorescence intensity increased with the numbers of PCR cycle, and reached a maximum at 45 cycles. Thus 45 cycles of PCR reaction cycle was used.

Circular dichroism (CD) spectroscopy was a powerful tool and was usually applied into the study of DNA structure^61^. It was reported that the negative peak of 210 nm was the marker for triplex DNA^62^, thereby CD spectroscopy was used to investigate the structure information of Oligonucleotide5′-CGA GTC CGT GGT AGG GCA GGT TGG GGT GAC TAA AGA GAA GGA AGA GAA GAA GAA AGA AAA GAA AAA GTT TTT TTG GCT TGT GGA GAG TCG TAC TGC AAT CGG GTA TT-3′ (R1) and Oligonucleotide5′-AAT ACC CGA TTG CAG TAC GAC TCT CCA CAA GCC AAA AAA ACT TTT TCT TTT CTT TCT TCT TCT CTT CCT TCT CTT TAG TCA CCC CAA CCT GCC CTA CCA CGG ACT CG-3′ (R2), which were two sequences of the PCR product. As shown in Fig. 2B, there were no (or little) negative peak at 210 nm for CD spectroscopy of R1·R2 duplex and MB respectively, while there occurred a strong negative peak at 210 nm for the mixture of R1·R2 and MB, which indicated that there was triplex formation between R1-R2 (PCR product) and MB. These results was in accordance with that of literature^49^,^50^,^51^,^52^,^53^,^54^.

**Figure 3:**
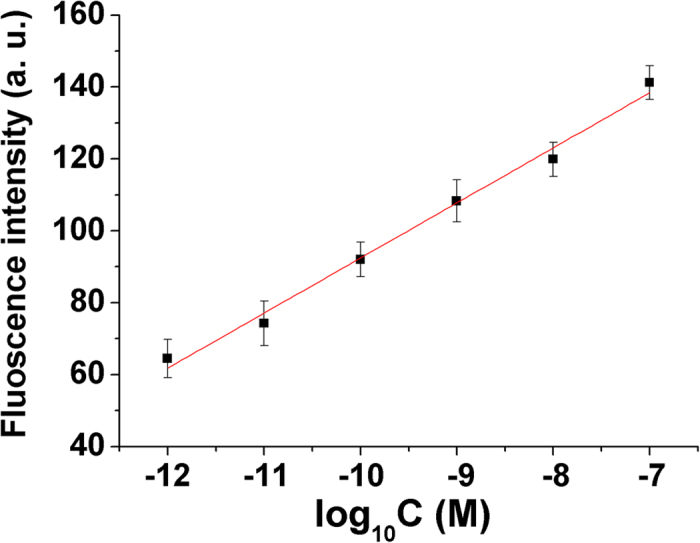
Calibration curve for the biosensor. 45 cycles of PCR amplification was carried out by using 30 s of denaturation at 94 °C, 30 s of annealing temperature at 60 °C, and 10 s of extending temperature at 72 °C. MB: 200 nM, spermine: 0.4 mM, pH:5.0. Each point was the mean of three measurements. The error bars are the standard deviation.

The sensitivity of the proposed strategy was investigated under the conditions of 200 nM MB, 0.4 mM of spermine, pH 5.0 and 90 minutes of hybridization time for fluorescence measurement, and 45 circles of PCR. As demonstrated in Fig. 3, the fluorescence intensity was proportional to the logarithm of the concentration of thrombin over the range from 1.0 × 10^−12^ M to 1.0 × 10^−7^ M. The linear regression equation was I = 15.12 log_10_*C* + 243.9 (C: M, r = 0.995, C represents the concentration of thrombin, I denotes the fluorescence intensity), with a detection limit of 261 fM, which was obtained from the equation of DL = 3б/slope. The comparison of the proposed method with others was listed in Table 1, it had merit of wide linear range.

**Figure 4:**
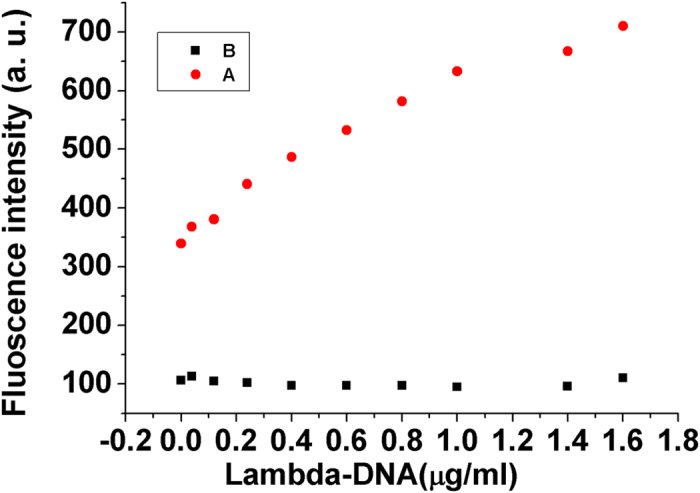
Effect of lambda - DNA on the fluorescence of the PCR product of 1.0 × 10^−8^ M thrombin. (**A**) Detecting PCR product by using SYRB - Green; SYRB - Green: 1 × , spermine: 0.4 mM, pH:5.0. (**B**) Detecting PCR product with the proposed method (detecting PCR product through triplex formation); MB: 200 nM, spermine:0.4 mM, pH:5.0.

Non - specific PCR amplification usually accompanied with the PCR amplification process. DNA contamination might easily occurred during PCR amplification process as well. Both DNA contamination and non - specific amplification PCR product were double - stranded DNA, which was similar to that of PCR product, so it was difficult to distinguish the signal for non - specific amplification and DNA contamination from that for PCR product, and then they usually affect the detecting result. To investigate the effect of non - specific PCR amplification and DNA contamination on the proposed strategy, the fluorescence intensity of the PCR product were measured with the proposed strategy and the common probe (SYRB - Green) for real - time PCR respectively. Here lambda - DNA was used to act as non - specific amplification product and DNA contamination due to its duplex structure. As shown in Fig. 4, the fluorescence intensity increased with concentration of lambda - DNA by using the probe of SYRB - Green, while the fluorescence intensity had little change when the concentration of lambda - DNA increased from 0 to 1.6 μg/mL by using the proposed strategy. These results indicated that the proposed strategy could overcome the effect of non - specific PCR amplification and DNA contamination completely, and the effect of non - specific amplification could be ignored in the proposed method. These results mainly due to the stringent sequence - specific recognition between PCR product and MB through triplex formation^49^,^50^,^51^,^52^,^53^,^54^.

**Figure 5:**
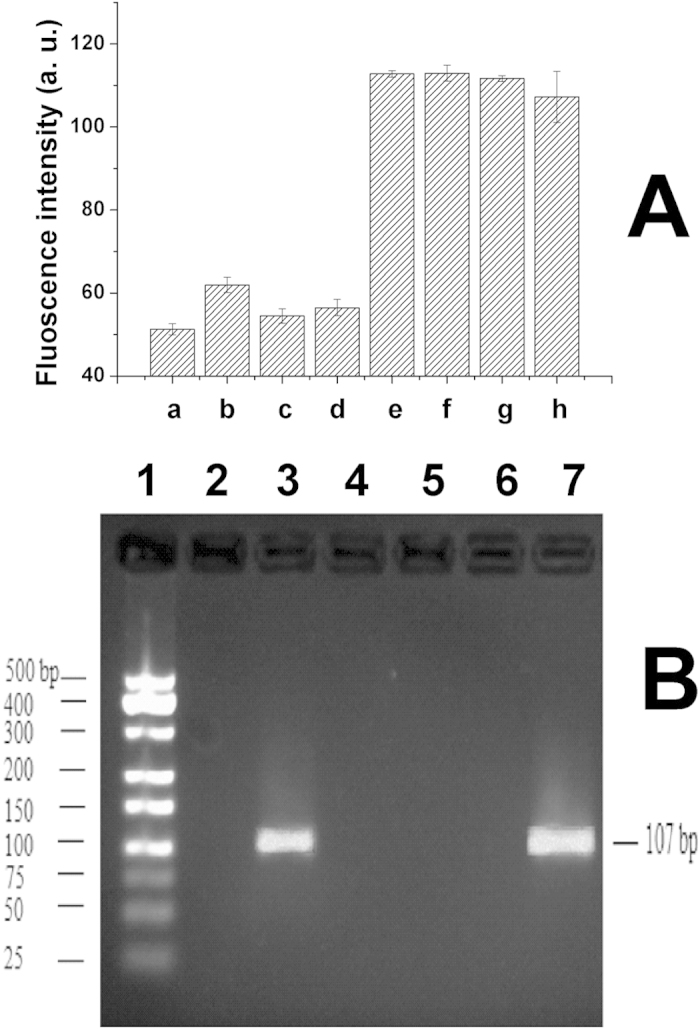
Selectivity of the sensor and effect of interference molecules. (**A**) a: blank; b: 1.0 × 10^−7^ M of lysozyme; c: 1.0 × 10^−7^ M of hemoglobin; d: 1.0 × 10^−7^ M of apo-transferrin human; e: 1.0 × 10^−8^ M of thrombin; f: mixture of 1.0 × 10^−8^ M of thrombin and 1.0 × 10^−7^ M of lysozyme; g: mixture of 1.0 × 10^−8^ M of thrombin and 1.0 × 10^−7^ M of hemoglobin; h: mixture of 1.0 × 10^−8^ M of thrombin and 1.0 × 10^−7^ M of apo-transferrin human. Every point was the mean of three measurements, error bar was the standard deviation. (**B**) Electrophoresis photograph of PCR product. 1. 500 bp DNA ladder; 2. Blank; 3. 1.0 × 10^−8^ M of thrombin; 4. 1.0 × 10^−7^ M of lysozyme; 5. 1.0 × 10^−7^ M of hemoglobin; 6. 1.0 × 10^−7^ M of apo-transferrin human; 7. mixture of 1.0 × 10^−8^ M of thrombin and1.0 × 10^−7^ M of lysozyme, hemoglobin, apo-transferrin human; EB: 0.5 μg/mL; Agarose: 3%.

In order to test the selectivity of the proposed sensor, the effect of other possible interferences were investigated. 1.0 × 10^−7^ M of lysozyme, hemoglobin and apo - transferrin human were used to replace the 1.0 × 10^−8^ M of thrombin for PCR amplification respectively, As demonstrated in Fig. 5A, each of their fluorescence intensity was almost at the same level of the blank. Moreover, the existence of 10-folded lysozyme, hemoglobin and apo -transferrin human had little effect on the fluorescence intensity of thrombin. To further certificate the selectivity of the sensor, electrophoresis photograph of PCR product was investigated, as demonstrated in Fig. 5B, there were no bands with addition of lysozyme, hemoglobin and apo-transferrin human, while there was the correct size band in the thrombin, which indicated that these proteins had no effect on detection of thrombin. These results indicated that the proposed biosensor had good selectivity.

To assess the analytical application of the sensor, the method was used to detect thrombin in human sera. Since no thrombin was found from the human sera, addition and recovery experiment was performed to estimate the application of the assay in complex sample. As demonstrated in Table 2, 5.0 × 10^−11^ – 5.0 × 10^−8^ M of thrombin was added into each human sera, the recovery ranges from 87.6% to 112.6%, and the relative standard deviation values were in the ranges of 4.3%–9.8%, which indicated that the method had good analytical application in human sera.

In summary, we have developed an ultrasensitive and selective sensor for thrombin by combining the PCR amplification and triplex formation. The fluorescence intensity was proportional to the logarithm of the concentration of thrombin over the range of 1.0 × 10^−12^ M–1.0 × 10^−7^ M, with a detection limit of 261 fM. The most important contribution is that the proposed strategy can overcome the effect of non - specific amplification and DNA contamination completely, which was realized by detecting PCR product with MB through triplex formation. Moreover, the proposed strategy may open a new avenue for the application of triplex DNA, it may be extend to immune - PCR and other aptasensor in the future.

## Methods

### Materials and Reagents

All Oligonucleotides were purchased from Sangon Bioengineering Technology and Services Co. Ltd. (Shanghai, China). Human thrombin, lysozyme (LYS), apo-transferrin human (ATH), hemoglobin (HB), spermine were purchased from Sigma-Aldrich (USA). Klenow Fragment (exo-) polymerase was purchased from Thermo scientific Inc (USA). Taq DNA Polymerase and dNTPs were obtained from Sangon Bioengineering Technology and Services Co. Ltd. (Shanghai, China). SYBR Green I (20×) was purchased from Bio Teke Corporation (Beijing, China). Nanopure water (18.1 MΩ) was obtained from a 350 Nanopure water system (Guangzhou Crystalline Resource Desalination of Sea Water and Treatment Co. Ltd.) and was used in all experiments. All chemicals were analytical grade unless stated.

Tris-HAc buffer (20 mM, pH 7.9, 50 mM KAc, 10 mM Mg (Ac)_2_, and 1.0 mM DTT) was used throughout the recognition of thrombin with aptamer. Phosphate buffered solution (PBS) (pH 5.0, 0.1 M Na_2_HPO_4_, 0.1 M NaH_2_PO_4_, and 0.1 M NaAc) was used for triplex formation between PCR product and MB.

### Apparatus

The PCR amplification was performed on a ETC 811 PCR Instrument (Eastwin Life Sciences, Inc., China). The fluorescence signal was measured on a RF-5301PC spectrofluorimeter (Shimadzu, Japan). The circular dichroism (CD) spectroscopy was obtained from a J-810-150S spectropolarimeter (JASCO International Co. Ltd., Japan).

### Recognition of thrombin and preparation of PCR template

Probe A and probe B were denatured at 90 °C for 5 min, then cooled to 0 °C rapidly. 2 μL of thrombin solution was mixed with 2 μL 4.0 × 10^−10^ M probe A and probe B in Tris-HAc buffer, kept at 37 °C for 30 min. After that, 0.5 U Klenow Fragment (exo-) polymerase and 0.9 μL 2.5 mM dNTPs were added, and the total mixture volume reached 10 μL, kept the mixture at 37 °C for 30 min, along with heating to 95 °C to inactivate the Klenow Fragment (exo-) polymerase. Then the inactivated mixture was acted as PCR template for following PCR amplification.

### PCR amplification

The PCR amplification was carried out in a 200 μL PCR tube that containing 5.0 μL PCR template, 2.0 μL 10 μM of two primers, 5 μL of 10 × PCR Buffer, 1 μL 5 U/μL of Taq DNA Polymerase, 3 μL 25 mM of MgCl_2_, 1 μL 10 mM of dNTPs and 31 μL of Nanopure H_2_O. The thermal program was comprised of an initial denaturation at 95 °C for 10 min, 45 cycles of PCR amplification was carried out by using 30 s of denaturation at 94 °C, 30 s of annealing temperature at 60 °C, and 10 s of extending temperature at 72 °C. All the reactions were run in triplicates, and the control experiment was carried out with same reaction mixture in the absence of thrombin.

### Measurement of fluorescence spectrum

10 μL of 10 μΜ MB were mixed with 490 μL PBS buffer solution which contained different amounts of PCR product. After 1.5 hours of incubation(25 °C), the fluorescence signal was measured with spectrofluorimeter. Slit widths were both 5.0 nm, and the excitation and emission wavelengths were set at 495 and 518 nm, respectively.

### Measurement of CD spectroscopy

The circular dichroism (CD) spectroscopy was measured at room temperature and performed over the wavelength range from 200 to 300 nm in 0.1 cm path length cuvettes. The result was obtained by averaging 3 scans at the scanning rate of 100 nm per minute with a response time of 1.0 s and the bandwidth of 1.71 nm.

## Additional Information

**How to cite this article**: Li, Y. *et al.* Triplex DNA: A new platform for polymerase chain reaction - based biosensor. *Sci. Rep.* **5**, 13010; doi: 10.1038/srep13010 (2015).

## Figures and Tables

**Table 1:** Comparison of the proposed method with others.

**Method**	**Analytical range**	**LOD**	**Application to samples**	**Ref.**
Electrochemistry	0.5 pM–20 nM	0.15 pM	no	63
Fluorescence	10 pM–10 nM	8.06 pM	yes	26
Colorimetry	0.1 pg/mL–50.0 pg/mL	0.083 pg/mL	yes	64
Surface enhanced Raman scattering	0.1 nM–10 nM	20 pM	yes	65
Surface plasmon resonance	25 fM–2 pM	25 fM	no	66
The proposed method	1.0 pM–100 nM	261 fM	yes	This work

**Table 2:** Recoveries of thrombin from the spiked human serum samples.

**Serum samples**	**Added thrombin(M)**	**Founded thrombin(M)**	**Recovery(%)**	**Relative standard deviation (%)**
1	5.0 × 10^−11^	5.63 × 10^−11^	112.6	9.8
2	5.0 × 10^−10^	4.66 × 10^−10^	93.2	7.5
3	5.0 × 10^−9^	4.38 × 10^−9^	87.6	8.9
4	5.0 × 10^−8^	5.44 × 10^−8^	108.8	4.3

The values shown here are the average values from three measurements.
